# Novel Heterozygous Missense Variants in Diacylglycerol Kinase Epsilon and Complement Factor I: Potential Pathogenic Association With Atypical Hemolytic Uremic Syndrome

**DOI:** 10.7759/cureus.52633

**Published:** 2024-01-20

**Authors:** Omar K Abughanimeh, Muhamed Baljevic, Alex Nester

**Affiliations:** 1 Division of Hematology-Oncology, Department of Internal Medicine, University of Nebraska Medical Center, Omaha, USA; 2 Division of Hematology-Oncology, Department of Internal Medicine, Vanderbilt University Medical Center, Vanderbilt-Ingram Cancer Center, Nashville, USA

**Keywords:** thrombotic microangiopathy (tma), cfi, dgke, ahus, atypical hemolytic uremic syndrome

## Abstract

Hemolytic uremic syndrome (HUS) is a thrombotic microangiopathy (TMA), which copresents with microangiopathic hemolytic anemia, thrombocytopenia, and kidney injury. While typical HUS is normally preceded by infections such as Shiga-toxin-producing *Escherichia coli, *atypical HUS (aHUS) has a genetic component that leads to dysregulation of the alternative complement pathway. We report a case of a 69-year-old female who developed aHUS after undergoing an elective knee surgery. Genetic testing revealed novel mutations affecting diacylglycerol kinase epsilon (DGKE) protein and complement factor I (CFI) that were not reported before as pathogenic. The patient was treated with eculizumab, leading to the complete resolution of TMA with no lasting organ damage.

## Introduction

Atypical hemolytic uremic syndrome (aHUS) is a rare thrombotic microangiopathy (TMA) attributed to genetic alterations of the alternative complement pathway [1]. These genetic mutations can present at any age [2]. Mutations causing aHUS can lead to either loss of function (affecting factor H and factor I) or gain of function (affecting C3 and factor B) [3]. In rare cases, new mutations are found that affect non-complement-related proteins such as diacylglycerol kinase epsilon (DGKE), plasminogen, and inverted formin-2. Mutation in the DGKE gene is very rare and typically presents in the first year of life [3]. DGKE is a protein involved in metabolism by affecting cell signaling. It is also responsible for the modulation of protein kinase C (PKC) activity. Hence, mutations affecting DGKE can result in the activation of PKC, which stimulates prothrombotic factors including tissue factor and von Willebrand factor [3].

## Case presentation

**Figure 1:**
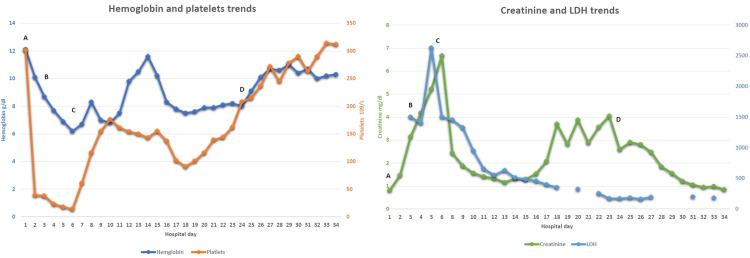
Trends of hemoglobin, platelets, LDH, and creatinine A: on the day of surgery; B: on the day of starting eculizumab; C: hemodialysis started; D: hemodialysis ended LDH: lactate dehydrogenase

A 69-year-old female with a history of hypertension, atrial fibrillation, chronic obstructive pulmonary disease (COPD), and chronic left knee septic joint following total knee arthroplasty, was admitted to the hospital for left knee cement spacer exchange. Even though the procedure was uncomplicated, she developed acute normocytic anemia (hemoglobin 8.7 g/dL), thrombocytopenia (53 x 10^3 ^µL), and acute kidney injury (creatinine 3.1 mg/dL) after three days of the surgery (Figure 1 shows the trend of relevant organ functions).

Further workup showed schistocytes on peripheral smear, elevated lactate dehydrogenase (LDH; 1540 U/L, reference range: 98-192 U/L), undetectable haptoglobin, total bilirubin of 0.5 g/dl (reference range: 0.2-1.5 mg/dL), international normalized ratio (INR) of 1.8, fibrinogen 239 mg/dL (reference range: 160-450 mg/dL). Otherwise, white blood cell counts, liver enzymes, and electrolytes were within normal limits. A disintegrin and metalloproteinase with a thrombospondin type 1 motif, member 13 (ADAMTS13) was sent given the concern of microangiopathic hemolytic anemias and the result showed 74% activity, suggesting atypical hemolytic uremic syndrome (aHUS).

The patient was started on eculizumab 900 mg weekly on the same day and hemodialysis after 48 hours (due to fluid overload and decreased urine output). Further studies revealed that the complement C3 level was 105 mg/dL (reference range: 90-180 mg/dL), complement C4 was 40 mg/dL (reference range: 12-45 mg/dL), CH50 was 87 CAE (reference range: 60-144 CAE), and complement factor B was 35.1 mg/dl. aHUS mutation panel revealed a heterozygous missense variant (c.562C>A (p.Pro188Thr)) in exon 3 of DGKE and a heterozygous missense variant (c.608C>T, p.Thr203Ile) in exon 4 of complement factor I (CFI). Her condition improved gradually on complement-directed therapy. She completed four weeks of weekly eculizumab followed by maintenance eculizumab 1200 mg every two weeks for six months. Her kidney function returned to normal levels after 24 days of complement-directed therapy, and she no longer required hemodialysis.

## Discussion

Recessive DGKE mutations causing TMA were first described in 2013 by Lemaire et al. [4]. In their study, they identified recessive mutations in DGKE that co-segregated with aHUS in nine individuals who were all aged less than one year at the time of diagnosis. Later, Mele et al. reported another novel mutation, which was the first to be located beyond the exon-intron boundaries [5]. Table 1 summarizes DGKE mutations in aHUS reported in the literature so far.

**Table 1:** Reported DGKE mutations in the literature in the English language DGKE: diacylglycerol kinase epsilon

DGKE mutations	Mutation type	Reference
c.32C>A p.Ser11*	Homozygous (premature termination codons)	Lemaire et al. [4]
c.472InsT p.Trp158Leufs*8	Homozygous (frameshift mutation)	Lemaire et al. [4]
c.818G>C p.Arg273Pro	Homozygous (missense mutation)	Lemaire et al. [4]
c.889-1G>A p.IVS5-1	Homozygous (splice site mutation)	Lemaire et al. [4]
c.486InsA p.Val163Serfs*3	Heterozygous (frameshift mutation)	Lemaire et al. [4]
c.188G>C p.Arg63Pro	Heterozygous (missense mutation)	Lemaire et al. [4]
c.966G>A p.Trp322*	Homozygous (premature termination codons)	Lemaire et al. [4]
c.1000C>T p.Gln334*	Homozygous (premature termination codons)	Lemaire et al. [4]
c.888+40A.G	Homozygous for one family/heterozygous for one family (splice site mutation)	Mele et al. [5]
c.231C>G, p.C77W	Heterozygous (missense mutation)	Li et al. [6]
c.790_791delTG, p.C264Yfs*27	Heterozygous (frameshift mutation)	Li et al. [6]
c.1213–2A>G	Heterozygous (splice site mutation)	Miyata et al. [7]
c.71delT	Heterozygous (frameshift mutation)	Miyata et al. [7]
p.(Phe250Serfs*3)	Homozygous (nonsense)	Alabdulqader et al. [8]
p.H536Qfs*16	Homozygous (frameshift mutation)	Sánchez Chinchilla et al. [9]
p.W322*/p.P498R	Heterozygous	Sánchez Chinchilla et al. [9]
p.Q248H/p.G484Gfs*10	Heterozygous	Sánchez Chinchilla et al. [9]
c.562C>A p.Pro188Thr	Heterozygous (missense mutation)	Our case

There is no sufficient evidence to recommend an optimal management method for DGKE mutation-induced aHUS. There are some reported cases where patients responded to either eculizumab or plasma exchange while in others they did not [10]. Our patient was further found to have a heterozygous missense variant (c.608C>T, p.Thr203Ile) in exon 4 of CFI. In general, CFI mutations are rarely reported in the literature to be associated with aHUS [11]. Almalki et al. reported a case of aHUS that was believed to be caused by a heterozygous CFI variant, c.944G > A (p.Arg315Lys) [11]. The CFI mutation in our case had never been reported in an aHUS previously, while some other reports have proposed a possible link with age-related macular degeneration. [12,13]

While recognizing that aHUS pathogenic variants in DGKE are indeed typically inherited in an autosomal recessive or compound heterozygote manner (and that most heterozygotes are usually asymptomatic), our patient’s clinical presentation was in the setting of (a) chronic septic arthritis where secondary causes of TMA were not evident after an exhaustive infections workup (no evidence of fungemia or bacteremia with three separate blood cultures negative over seven days); (b) negative medical history for drug-induced causes; (c) only notable finding among a mutation profile that screened more than 20 different coagulation pathway genes coding for proteins known to be pathogenically associated with aHUS were variants of unknown significance in DGKE and CFI reported in this case. Unfortunately, more definitive data regarding each variant (e.g., minor allele frequency and in silico analysis) could not be provided due to technical limitations. Lastly, the dramatic clinical improvement following anti-complement therapy as well as the presence of heterozygous mutations in two genes known to be pathogenically involved with aHUS led us to postulate that either, alone or in combination as in this case, may be pathogenically relevant. 

In general, aHUS has a poor prognosis with around 60% of patients progressing to end-stage renal disease (ESRD). It has an estimated mortality rate of 4-25% [5]. In this instance, complement-directed therapy was effective and prevented conditions associated with long-term organ damage such as ESRD. Considering the brisk clinical response to therapeutic challenge with anti-complement therapy, as well as previously noted associations of DGKE and CFI variants with aHUS, we believe that variants of unknown significance in this case potentially played a pathogenic role, and should be further examined for pathogenic potential at centers with adequate capabilities for minor allele frequency and in silico analysis, during subsequent screening efforts for patients with suspected aHUS.

## Conclusions

aHUS is a rare, life-threatening disease with a genetic component. Mutations in DGKE are rare and usually present in the first years of life, though exceptions in the elderly can be found, leading to aHUS. Similarly, CFI mutations leading to aHUS are not commonly reported in the literature. There is currently no standard treatment for aHUS caused by DGKE or CFI mutations, but our case was successfully treated with eculizumab therapy.
